# Elevated stress response marks deeply quiescent reserve cells of gastric chief cells

**DOI:** 10.1038/s42003-023-05550-2

**Published:** 2023-11-20

**Authors:** Daisuke Shiokawa, Hiroaki Sakai, Miho Koizumi, Yoshie Okimoto, Yutaro Mori, Yusuke Kanda, Hirokazu Ohata, Hiroaki Honda, Koji Okamoto

**Affiliations:** 1 Division of Molecular Pharmacology, National Cancer Center Research Institute, 5-1-1 Tsukiji, Chuo-ku, Tokyo 104-0045 Japan; 2 Ehime University Hospital Translational Research Center, Shitsukawa, Toon, 791-0295 Ehime Japan; 3 Advanced Comprehensive Research Organization, Teikyo University, 2-21-1 Kaga, Itabashi-ku, Tokyo 173-0003 Japan; 4 Field of Human Disease Models, Major in Advanced Life Sciences and Medicine, Tokyo Women’s Medical University, 81- Kawada-cho, Shinjuku-ku, 162-8666 Tokyo Japan

**Keywords:** Gastrointestinal models, Cell-cycle exit

## Abstract

Gastrointestinal tract organs harbor reserve cells, which are endowed with cellular plasticity and regenerate functional units in response to tissue damage. However, whether the reserve cells in gastrointestinal tract exist as long-term quiescent cells remain incompletely understood. In the present study, we systematically examine H2b-GFP label-retaining cells and identify a long-term slow-cycling population in the gastric corpus but not in other gastrointestinal organs. The label-retaining cells, which reside near the basal layers of the corpus, comprise a subpopulation of chief cells. The identified quiescent cells exhibit induction of Atf4 and its target genes including *Atf3*, a marker of paligenosis, and activation of the unfolded protein response, but do not show elevated expression of *Troy*, *Lgr5*, or *Mist*. External damage to the gastric mucosa induced by indomethacin treatment triggers proliferation of the quiescent Atf4^+^ population, indicating that the gastric corpus harbors a specific cell population that is primed to facilitate stomach regeneration.

## Abstract (web summary)

Systematic examination of H2b-GFP label retaining cells in mouse digestive organ uncovered a slow-cycling subpopulation of gastric chief cells that uniquely express markers of paligenosis and are ready to undergo proliferation after external damage.

## Introduction

Hierarchically organized tissues such as hematopoietic tissues, skeletal muscle, neuronal tissues, and hair follicles harbor quiescent cells as a source for regeneration of damaged tissues if incumbent stem cells are damaged or lost due to external insults^1,2^. The quiescent reserved cells in these organs, which often exhibit stem cell-like characteristics, maintain resident stem cell populations and organ homeostasis.

The gastrointestinal tract, which constantly faces direct environmental exposure, is also equipped with reserve cells to repair its damage. The process of regeneration after injury has been extensively studied in the small intestine of the lower gastrointestinal tract. In the absemce of damage, *Lgr5*^*+*^ actively-cycling stem cells, which are present at the bottoms of intestinal crypts, differentiate into all lineages in the crypt structure. In response to severe tissue damage, slow-cycling cells that are located at the +4 position are converted into fast-cycling *Lgr5*^*+*^ stem cells^3^. These observations suggest that +4 cells serve as cellular reservoirs of stem cells^4–6^. Alternatively, the conversion of the +4 cells into stem cells could be interpreted as dedifferentiation of progenitor cells^7,8^.

In the upper gastrointestinal tract, tissue regeneration after injury has been mainly studied in the stomach. The stomach is divided into several anatomically distinct regions, including the antrum and corpus^9^. The gastric units in the antrum and corpus both have an isthmus region that is the source of bidirectional proliferation of stem cells^9^. As is the intestine, *Lgr5*^*+*^ cells reside at both the gland base and +4 position in the antrum, and the expression of *Lgr5* or *Aqp5* in the gland base marks cycling stem cell populations.

Notably, in the corpus region, chief cells are an additional source of reserve cells. Chief cells are slow cycling and function as a reserve for regeneration of the gastric unit after severe damage^10,11^. Of note, lineage tracing assays have indicated that several chief cell-expressed genes, including *Lgr5*, *Troy*, *Mist1*, *Gpr30*, *GIF*, and *p57*, behave as markers of damage-responsive reserve cells^12–17^. Although these reports have demonstrated the plasticity of chief cells, the molecular basis of this phenomenon remains unclear.

Interestingly, during the regeneration, chief cells can undergo dramatic reprogramming resulting in a type of metaplasia known as spasmolytic polypeptide–expressing metaplasia (SPEM)^10,17–20^. This metaplastic response is called paligenosis^21^, and has recently attracted attention as it was proposed that gastric cancer arises from the resulting metaplasia^20^. The dynamic process of paligenosis involves several stages, and transient inhibition of mTORC1 and activation of the autophagic/lysosomal response are followed by induction of metaplastic genes, including *Atf3*^22^ or *Sox9*^23^.

In this paper, we systematically searched for slow-cycling reserve cells in the epithelia of digestive organs. Previously, H2B-GFP label-retaining assays have been successfully applied to identify quiescent cells in diverse organs^24–29^. Therefore, to evaluate the presence of quiescent reserve cells in the gastrointestinal tract, we performed a label-retaining assay after temporary H2b-GFP expression. Remarkably, we detected GFP^+^ epithelial cells for >2 months after labeling in the gastric corpus but not in other digestive tract organs. Expression analyses demonstrated that the GFP^+^ subpopulation of chief cells specifically expressed Atf4. Furthermore, these GFP^+^ cells started proliferation after tissue damage, indicating that the identified quiescent cells share phenotypic similarity with cells undergoing paligenosis upon tissue damage.

## Results

### Gastric epithelia specifically harbor label-retaining long-term quiescent cells

**Fig. 1:**
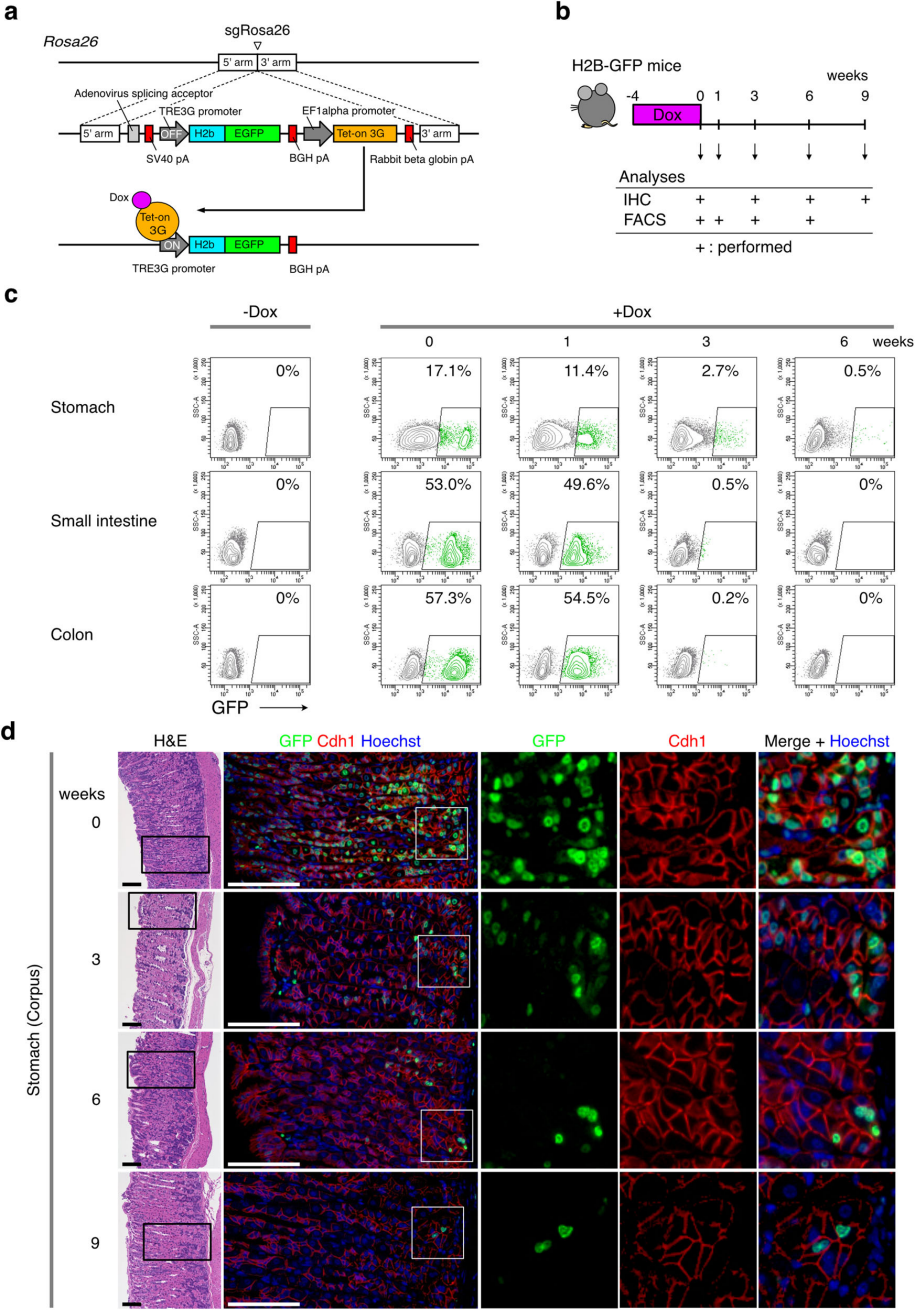
Stomach epithelia specifically harbor label-retaining long-term quiescent cells. **a** Genomic structure of the *Rosa26* loci after insertion of the inducible H2b-GFP and Tet-on 3 G cassettes. Constitutively expressed Tet-on 3 G activates the TRE3G promoter to express the H2b-GFP gene in a doxycycline (Dox)-dependent manner. The cassette was chemically synthesized with flanking 5’ and 3’ sequences and inserted in the *Rosa26* locus by sgRNA-guided homologous recombination. The open arrowhead indicates the sgRNA targeting site. **b** Experimental scheme for time course analysis of the H2b-GFP-labeled cells in the digestive tissues. Four-week-old mice were treated with doxycycline for 4 weeks and examined at the indicated time points by immunohistochemistry and/or FACS. **c** Flow cytometry analysis of the GFP^+^ cells in epithelia of the indicated digestive tissues. **d** Hematoxylin and eosin (H&E) (upper panel) and immunostaining (lower panel) of the H2b-GFP^+^ cells in the gastric corpus. Black boxes indicate the regions shown in immunostaining. Magnified images of white boxes are shown at bottom column. Scale bar, 100 µm.

To investigate the presence of long-term quiescent cells in the gastrointestinal tract, we performed long-term labeling with an inducible chimeric mouse histone 2B-green fluorescent protein (H2b-GFP). To do this, H2b-GFP regulated by a tetracycline operator (TRE3G) was knocked in at the *Rosa26* locus together with a third-generation tetracycline-inducible transactivator (Tet-on 3 G)^30^ to generate Rosa26^iH2b-GFP^ mice (Fig. 1a and Supplementary Fig. 1a). In Rosa26^iH2b-GFP^ mice, the fusion protein was widely induced in the gastrointestinal epithelia after oral *ad libitum* administration of doxycycline. Rosa26^iH2b-GFP^ mice were subjected to H2b-GFP pulse-labeling for 4 weeks, which was followed by chase periods in the absence of doxycycline (1–9 weeks). Subsequently, the gastrointestinal tracts of the labeled mice were examined by immunostaining and flow cytometry (Fig. 1b and Supplementary Fig. 1b).

Flow cytometric analyses of the stomach, small intestine, and colon indicated that Epcam^+^ epithelial cells in these organs were pulse-labeled with H2b-GFP with varying frequencies (stomach: 17.1%, small intestine: 53.0%, colon: 57.3%) (Fig. 1c, 0 weeks after doxycycline treatment). In the small intestine and colon, cell proliferation caused rapid loss of GFP signals in the GFP^+^ population, and GFP^+^ cells were nearly undetectable within 3 weeks. In contrast, in the stomach, 2.9% of GFP^+^ cells (0.5% out of the initially labeled population (17.1%)) retained detectable GFP signals after 6 weeks (Fig. 1c).

To validate the unique retention of pulse-labeled H2b-GFP in the stomach epithelial subpopulation, we examined retained H2b-GFP expression in multiple gastrointestinal organs using immunostaining. Remarkably, GFP^+^ cells were detected in the lower area of the gastric corpus even 9 weeks after the start of the chase periods (Fig. 1d). The GFP-labeled cells were E-Cadherin (Cdh1)-positive, indicating that these cells were of epithelial origin (Fig. 1d). In contrast, GFP-labeled cells were almost undetectable after 3 weeks in other gastrointestinal organs including the esophagus, forestomach, antrum, jejunum, and colon (Supplementary Fig. 1c), indicating that the presence of long-term quiescent cells in the stomach was unique among organs of the gastrointestinal tract.

### Deeply quiescent cells in the stomach are localized in the gastric corpus

**Fig. 2:**
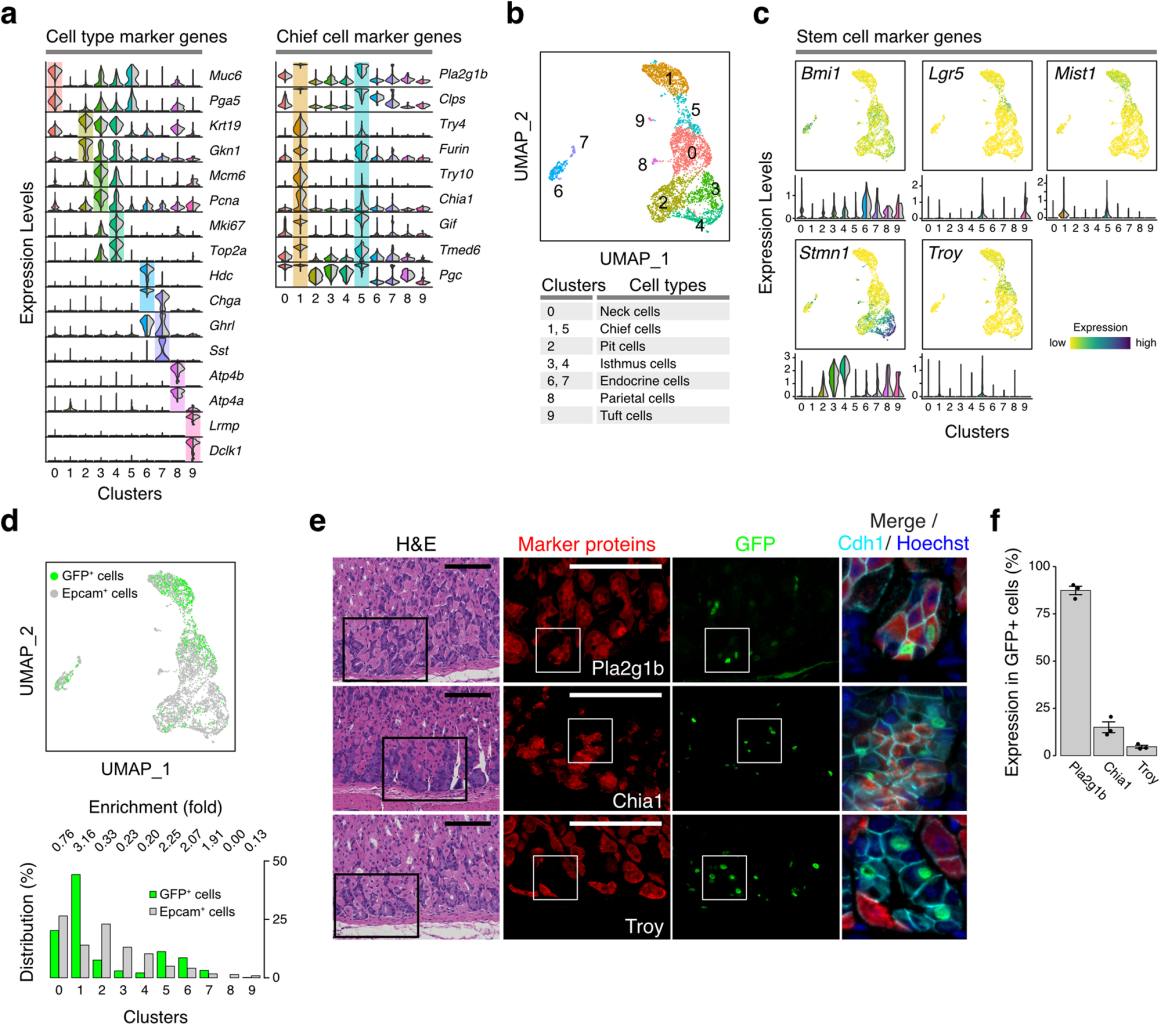
Long-term quiescent cells in the stomach are localized to the gastric corpus. **a** Expression profiles of cell type marker genes of the stomach in each cluster (clusters 0-9). 6 weeks after the pulse-chase, stomach epithelial cells (Epcam^+^ cells) were subjected to FACS-sorting to isolate GFP^+^ cells. A mixture of Epcam^+^ cells and GFP^+^ cells was used to perform single-cell RNA-seq and classified into ten subpopulations. Gene expression profiles of the markers in each subpopulation are shown in the split violin plots (left: a mixture of Epcam^+^ cells and GFP^+^ cells, right: Epcam^+^ cells). The cellular identity of each cluster was determined based upon expression of Chief cell markers (right panel) and other cell-type specific markers (left panel). **b** Two-dimensional projection of each subpopulation with UMAP (upper panel). The cell type of each subpopulation shown in (a) is summarized (lower panel). **c** Expression levels of stem cell marker genes in the stomach epithelial cells shown in UMAP Feature plots (upper panels) and split violin plots (lower panels). (left: a mixture of Epcam^+^ cells and GFP^+^ cells, right: Epcam^+^ cells). **d** A UMAP projection of the GFP^+^ cells (green) and Epcam^+^ cells (gray). The relative distributions of GFP^+^ cells and Epcam^+^ cells are shown in lower panel. The relative ratio of GFP^+^ cell numbers and Epcam^+^ cell numbers in each cluster are represented as fold enrichment values. **e** H&E and immunostaining of the H2b-GFP^+^ cells in the stomach corpus following a 6-week chase period. Black boxes in the H&E images indicate the regions corresponding to the immunostainings (Marker proteins and GFP). Scale bar, 100 µm. Areas used for merged images (far right panels) are shown as white squares. **f** Quantification of a fraction of GFP^+^ cells in cells that express the indicated cell-type markers. The quantified values are presented as the mean ± SD. n = 3 biologically independent experiments.

To identify the cell-types associated with the GFP^+^ label-retaining corpus epithelial cells, we performed single-cell RNA-seq analyses of FACS-sorted Epcam^+^ cells and Epcam-positive GFP^+^ cells (GFP^+^ cells) (Supplementary Fig. 1b). Based upon gene expression profiles, the Epcam^+^ cells were stratified into 10 subpopulations (Fig. 2a). When we combined the GFP^+^ cells with the Epcam^+^ cells (mixed at a ratio of ~4.5 to 1; hereafter called GFP-mixed cells), we could stratify the mixture into the same 10 subpopulations (Fig. 2a), which allowed us to evaluate the cell types of the GFP^+^ cells in the following studies.

The cellular identity of each subpopulation was determined based upon the expression of representative cell type markers and cell cycle markers (Fig. 2a–c and Supplementary Fig. 2a)^31–35^. Isthmus subpopulations (clusters 3 and 4) were identified by elevated expression of *Mki67* and cell cycle markers (*Pcna*, *Mcm6*, and *Top2a*) (Fig. 2a, left panel and Supplementary Fig. 2a). Unique expression of cell cycle markers indicated that isthmus cells were the only actively proliferating subpopulations. The chief cell subpopulations (clusters 1 and 5) were marked by the expression of *Pla2g1b*, *Clps*, *Furin*, *Chia1*, *Gif*, and *Tmed6*^31^ (Fig. 2a, right panel and Supplementary Fig. 2a). We also detected digestive enzyme-encoding genes that were preferentially expressed in the cluster 1 subpopulation (*Try4* and *Try10*) (Fig. 2a, right panel and Supplementary Fig. 2a).

Chief cells were reported to harbor quiescent stem cells with distinct markers^33^. Indeed, *Lgr5* and *Troy* were highly expressed in cluster 5, and *Mist1* was highly expressed in both clusters 1 and 5. As expected, *Bmi1* and *Stmn1*, markers of isthmus stem cells^11,36^, were not highly expressed in either cluster 1 or 5 (Fig. 2c).

We subsequently determined whether the GFP^+^ cells could be classified into distinct subpopulations. The GFP^+^ cells were preferentially enriched in cluster 1 (Fig. 2d). Coimmunostaining indicated that the GFP^+^ cells near the basal layers did not express markers specific for isthmus cells (Mki67), Pit cells (Krt19), Tuft cells (Dclk1), or endocrine cells (Chga) (Supplementary Fig. 2b) but did express Pla2g1b, a chief cell marker (Fig. 2e, f). These data indicate that the GFP^+^ cells near the basal layers were primarily classified as the cluster 1 subpopulation of chief cells.

### Atf4 and the unfolded protein response pathway are activated in long-term quiescent chief cells

**Fig. 3:**
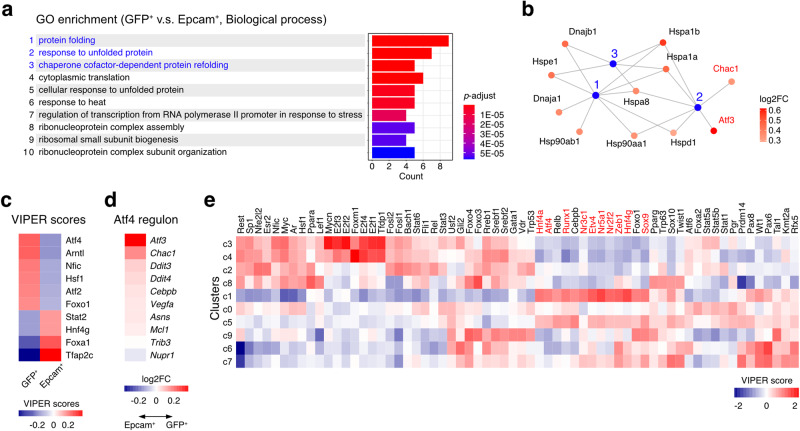
Atf4 and the unfolded protein response pathway are activated in deeply quiescent chief cells. **a** Gene Ontology (GO) term enrichment analyses (biological process) of the GFP^+^ chief cells. The *p*-values and gene counts of top ten GO terms enriched in GFP^+^ cells in comparison to Epcam^+^ cells are shown. The top three terms were shown in blue characters. The *p* value of each GO term is color-labeled. **b** A gene concept network for the GO terms enriched in GFP^+^ cells. Blue dots represent the top three terms shown in **a** (1, protein folding: 2, response to unfolded protein: 3, chaperone cofactor-dependent protein refolding). Genes that were associated with these terms were shown. Genes belonging to Atf4 regulon (Atf3, Chac1) are shown in red. Extent of induction of these genes in GFP^+^ cells are shown as color shading. **c** The top ten transcription factors that were differentially activated between GFP^+^ and Epcam^+^ cells. VIPER activation scores of each transcription factor in GFP^+^ and Epcam^+^ cells are color-coded. **d** Upregulation of the indicated Atf4 regulon genes in GFP^+^ cells relative to Epcam^+^ cells. The Atf4 regulon is composed of 16 genes and the comparison of the regulon genes with detectable expression is shown. Relative expression ratios are shown as log_2_FC values**. e** VIPER scores of differentially expressed transcription factors in each cluster. The data matrix was sorted by hierarchical clustering (complete linkage method) on both axes and represented as a heatmap. Transcription factors upregulated in the c1 cluster are indicated in red.

Subsequently, we compared the transcriptomes of Epcam^+^ and GFP^+^ cells from cluster 1 by using single-cell RNA-seq data to investigate biological signatures associated with the GFP^+^ cells. Gene Ontology (GO) term enrichment analyses indicated that the GFP^+^ cell-associated gene signature included unfolded protein response (Fig. 3a and Supplementary Fig. 3a). Identification of genes shared among the signatures revealed the existence of a complex network of heat shock-related genes whose expression levels were elevated in the GFP^+^ cells (Fig. 3b and Supplementary Fig. 3b). In accordance with these data, single-sample GSEA indicated that the unfolded protein response gene signature was highly expressed in quiescent GFP^+^ cells (Supplementary Fig. 3c).

In addition, computational inference of transcription factor activity by VIPER algorithm^37^ revealed that the transcriptional activity of Atf4, a key mediator of the integrated stress response^38,39^, was markedly increased in GFP^+^ cells (Fig. 3c). Accordingly, most Atf4 downstream target genes were upregulated in GFP^+^ cells (Fig. 3d). In addition to Atf4, several other transcription factors involved in the stress response (Hsf1, Atf2, Foxo1) were activated (Fig. 3c). Importantly, VIPER analyses of the ten stomach cell clusters (Fig. 2a, b) indicated that Atf4 was specifically activated in cluster 1 of the chief cells (Fig. 3e). These data indicated that activation of Atf4 and the unfolded protein response were unique hallmarks of the long-term quiescent GFP^+^ chief cells.

### Atf4^high^ quiescent cells comprise a unique subpopulation of chief cells

**Fig. 4:**
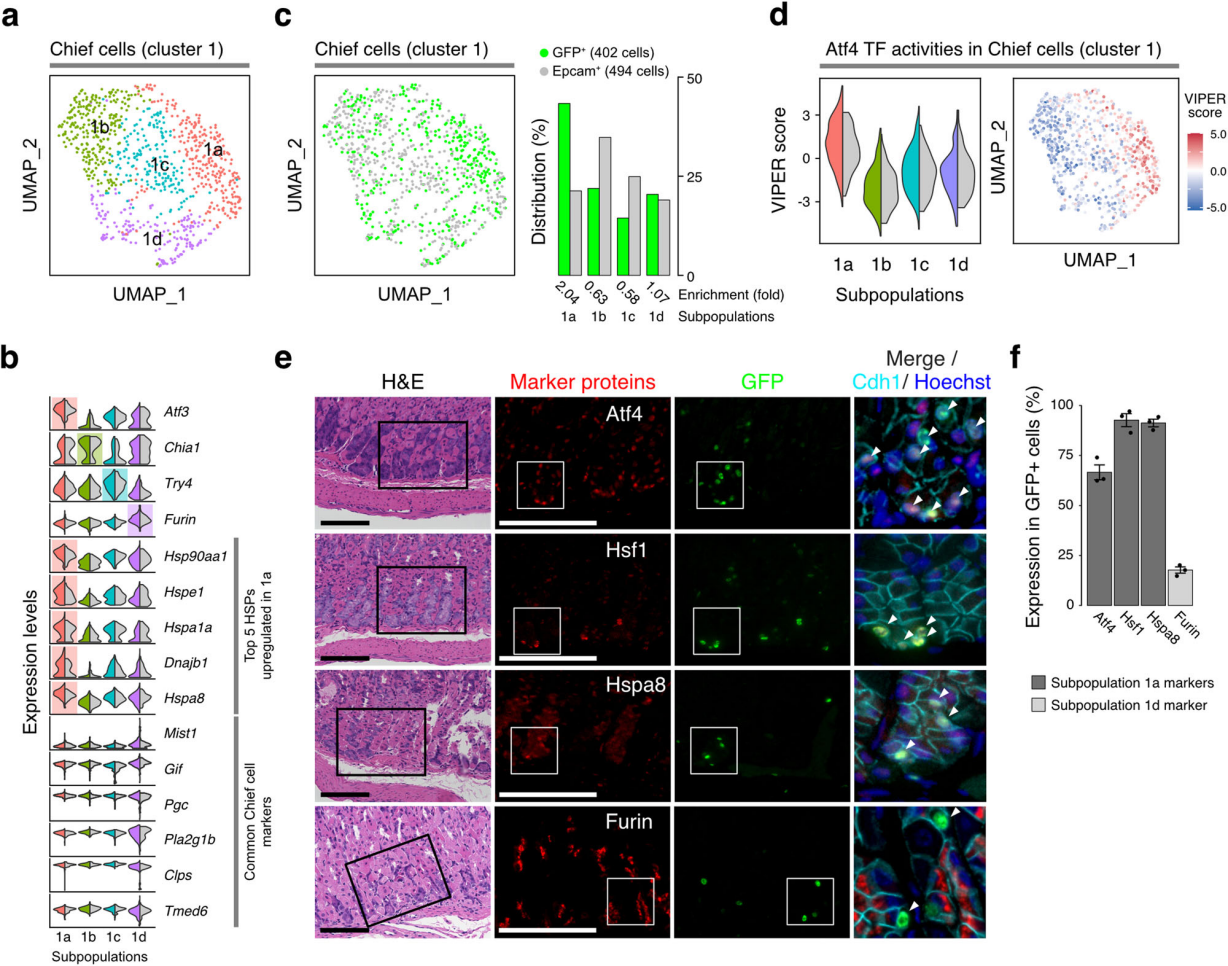
Atf4^high^ quiescent cells comprise a unique subpopulation of chief cells. **a** A UMAP projection of cluster 1 cell classification into four subpopulations. **b** Split violin plots in each subpopulation of known chief cell marker genes (*Mist1, Gif, Pgc*, *Furin*, *Pla2g1b*, *Clps*, and *Tmed6*) and the top 5 heat shock proteins (HSPs) upregulated in the 1a subpopulation (*Hsp90aa1, Hspe1, Hspa1a, Dnajb1*, and *Hspa8*) (left: a mixture of Epcam^+^ cells and GFP^+^ cells, right: Epcam^+^ cells). *Atf3*, a Atf4-regulated gene, is also shown as a marker of the Atf4^high^ cells. Genes that are preferentially expressed in each subpopulation of cluster 1 are highlighted. **c** A UMAP projection of the GFP^+^ cells (green) and Epcam^+^ cells (gray) in cluster 1 and relative distributions of GFP^+^ cells and Epcam^+^ cells (right panel). The relative ratios of GFP^+^ cell numbers and Epcam^+^ cell numbers in each cluster is shown as fold enrichment values. **d** Split violin plot of Atf4 VIPER value of each subpopulation of cluster 1 (left: a mixture of Epcam^+^ cells and GFP^+^ cells, right: Epcam^+^ cells). Feature plot presentation of the Atf4 VIPER value is shown at right panel. **e** H&E and immunostaining of the H2b-GFP^+^ cells in the stomach corpus at 6 weeks of post-chase periods. Black boxes indicate the regions used for immunostaining with the indicated antibodies. Scale bar, 100 µm. Areas used for merged images (far right panels) are shown as white squares. **f** Percentage fractions of H2b-GFP^+^ cells expressing the indicated markers shown in **e**. The GFP^+^ cells located within bottom third of the corpus are evaluated and presented as the mean ± SD. n = 3 biologically independent experiments.

To examine whether the GFP^+^ cells formed a distinct chief cell subpopulation, we further analyzed single-cell RNA-seq data of the cluster 1 population, in which GFP^+^ cells were highly enriched (Fig. 2d). We stratified the cluster 1 population of chief cells into four subpopulations (1a, 1b, 1c, and 1d) (Fig. 4a, b). Some chief cell markers were ubiquitously expressed in all subpopulations (*Pla2g1b*, *Clps*, and *Tmed6*), while others were preferentially expressed in one of the subpopulations (*Chia1* in 1b and *Furin* in 1d). Of note, a majority of GFP^+^ cells expressed Pla2g1b, but not Chia1 or Troy (Fig. 2e, f), indicating that a fraction of chief cell population constituted GFP^+^ cells.

Notably, the GFP^+^ cells were markedly enriched in the 1a subpopulation (Fig. 4c). In accordance with Atf4 activation and elevated heat shock gene expression in the GFP^+^ cells, elevated expression of the downstream targets of Atf4 (Fig. 4b; *Atf3* and Supplementary Fig. 4a) and heat shock genes (Fig. 4b) as well as preferential activation of Atf4 (Fig. 4d) were observed in the 1a subpopulation. In accordance with these findings, immunostaining revealed that most of the GFP^+^ cells near the basal layers expressed Atf4 and heat shock proteins (Hsf1, Hspa8) but not Furin (Fig. 4e). Furthermore, ssGSEA revealed preferential activation of the unfolded protein response in the 1a subpopulation (Supplementary Fig. 4b). These data indicated that the GFP^+^ chief cells were primarily classified into the 1a subpopulation, in which Atf4 and the unfolded protein response were activated.

Subsequently, we examined whether the 1a subpopulation of cluster 1 preferentially expresses chief cell markers. As expected, many of the Atf4 target genes were preferentially expressed in the 1a subpopulation (Fig. 4b, c, Supplementary Fig. 4a), and correlation analyses indicated that the expression of Atf4 target genes (*Atf3* and *Chac1*) was distinct from that of the other known markers (Supplementary Fig. 4d). In accordance with the association of GFP^+^ cells with the 1a subpopulation, *Atf3* was uniquely upregulated in GFP^+^ cells among chief cell markers (Supplementary Fig. 4e) and most GFP^+^ cells expressed Atf4 and heat shock proteins (Hsf1, Hspa8) but not Furin (Fig. 4e, f).

In contrast, *p57* (*Cdkn1c*), *Gpr30*, and *Mist1* did not show preferential expression among the subpopulations (Supplementary Fig. 4c). In addition, expression of *Lgr5* and *Troy* was hardly detectable in any of the subpopulations (Supplementary Fig. 4c), which is consistent with the lack of expression of these chief cell markers in cluster 1 (Fig. 2c). These data indicated that the Atf4^+^/GFP^+^ cells comprised a distinct quiescent population of chief cells.

### External insults initiate the proliferation of Atf4^high^ quiescent chief cells

**Fig. 5:**
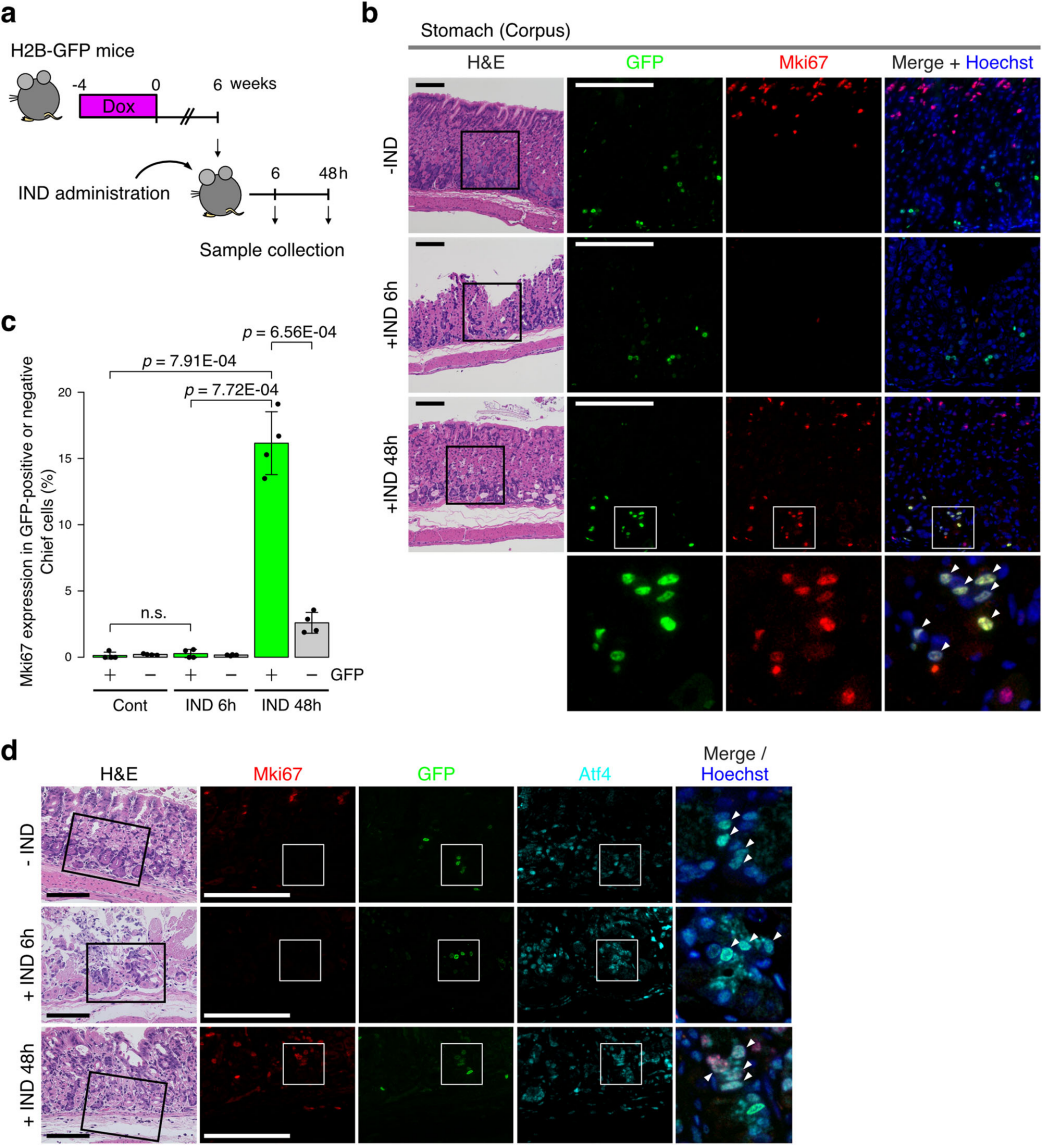
Atf4^high^ quiescent chief cells are capable of initiating proliferation in response to external insults. **a** Experimental scheme for Indomethacin (IND)-induced stomach damage models. Indomethacin was administered orally at 6 weeks after the start of post-chase periods and histological analyses was performed at 6 h or 48 h after IND treatment. **b** H&E and immunostaining of the H2b-GFP^+^ cells in the stomach corpus following indomethacin-induced injury. At 48 hr after indomethacin treatment, Mki67 expression was unevenly distributed in GFP^+^ cells probably due to variegated effects of the treatment, and representative images near the basal layers where GFP^+^ cells highly express Mki67 are shown. Magnified images of white boxes are shown at bottom column. Arrowheads indicate Mki67^+^/GFP^+^ cells. -IND: untreated control. Scale bar, 100 µm. **c** Average fraction of Mki67^+^ cells among GFP-positive and negative chief cells at the indicated time points after the IND-induced injury. Mki67 staining was evaluated in GFP^+^/ Pla2g1b^+^ cells and GFP^-^/ Pla2g1b^+^ cells. The quantifed values are presented as the mean ± SD. n.s., not significant. n = 4 biologically independent experiments. **d** Representative images of triple staining of Mki67, GFP, and Atf4 at the indicated time after indomethacin treatment are shown. Merged images of white boxes are shown at far right. Arrowheads indicate Mki67^+^/GFP^+^/Atf4^+^ cells. -IND: untreated control. Scale bar, 100 µm.

Finally, we sought to determine the potential function of GFP^+^ quiescent cells as cellular reserves for tissue restoration following external insults. Rosa26^iH2b-GFP^ mice were subjected to pulse-chase as in Fig. 1b (chase period: 6 weeks) and then orally administered with indomethacin to determine whether proliferation of the GFP^+^ cells was initiated in response to tissue injury (Fig. 5a). Indomethacin is a commonly used nonsteroidal anti-inflammatory drug that induces gastric lesions and apoptosis of the gastric mucosa^40^. As expected, indomethacin induced extensive destruction of gland structures in the stomach (Fig. 5b). Remarkably, while Mki67 expression was not detected in the GFP^+^ cells without indomethacin treatment, a substantial fraction of the surviving GFP^+^ cells (~20%) expressed Mki67 at 48 hr after treatment (Fig. 5b, c). In addition, a majority of Mki67-expressing GFP^+^ cells expressed Atf4 (Fig. 5d). This demonstrated that the long-term quiescent GFP^+^/Atf4^+^ cells were capable of initiating cell proliferation in response to external insults, suggesting that they could function as cellular reserves for damaged tissues.

## Discussion

In the present study, we systematically examined the gastrointestinal tract to identify long-term quiescent cells that retained H2b-GFP for extended periods in pulse-chase labeling experiments. We demonstrated that a subpopulation of chief cells in the gastric corpus remained in a slow-cycling state for > 2 months and yet were capable of initiating proliferation following tissue injury. Expression profiles of previously identified cell markers indicate that the identified population was distinct from previously reported reserve cell populations. In addition, the identified subpopulation exhibited a gene signature characterized by the unfolded protein response and activation of Atf4, a transcription factor associated with paligenosis. These results indicate that the identified slow-cycling cells represent a unique subset of chief cells that are associated with elevated stress response and primed for proliferation and tissue regeneration.

After tissue injury, the reserve cells are thought to transform into cycling cell population due to cellular plasticity^8^. The chief cell population, which is located near the basal layer of the corpus gland, is known to harbor reserve cell populations^33^. Burclaff et al. performed BrdU-labeling experiments and showed that chief cells, which rarely proliferate in the absence of damage, undergo massive proliferation after treatment with damage-inducing agents^10^. It is likely that the rarely proliferating BrdU-labeled cells that are present in the absence of damage correspond to the H2b-GFP labeled cells in this study. Here, we exploited live cell sorting of GFP-labeled cells to perform a molecular characterization of this rare population of the slow-cycling chief cells.

Previous reports have demonstrated that chief cell populations expressing markers, including *Lgr5*, *Troy*, *Mist1*, *Gpr30*, and *p57*, can regenerate the gastric corpus, indicating that cells expressing these markers function as a reservoir to reconstruct damaged tissues^12,13,41^. The expression of these markers was not associated with the GFP^+^-labeled population in our study, as *Mist1*, *Gpr30*, and *p57* were detectable at similar levels in four subpopulations of chief cells while *Lgr5* and *Troy* were undetetable in any of them. In contrast, Atf4 activity and the expression of its downstream genes were elevated in the 1a subpopulation. Considering that Atf4^+^/GFP^+^ cells preferentially start proliferation after injury, it is possible that Atf4 marks a subpopulation of chief cells that mainly contributes to tissue regeneration after indomethacin treatment.

Atf4 has been reported to be a key mediator of the integrated stress response^38,39^. Interestingly, the identified GFP^+^ cells were also associated with the unfolded protein response, which, together with Atf4, may confer higher resilience to damage-induced stress. Given their predicted anti-stress capacity, the GFP^+^ Atf4^high^ cells may preferentially survive during external injury.

Notably, induction of ATF3, a transcriptional target of Atf4, is one of the earliest events during the stepwise processes that mediate SPEM cell generation after injury^22^, suggesting that SPEM cells and the GFP^+^ cells share overlapping biological traits. In support of this possibility, another transcriptional target of Atf4 that was upregulated in GFP^+^ cells was *Ddit4* (Fig. 3d), which facilitates the initial phase of paligenosis via suppression of mTORC1^42^. In addition, one of the other transcription factors activated in the c1 subpopulation was Sox9 (Fig. 3e), which is another key player during the metaplastic processes of SPEM^21^. Considering the potential association of SPEM with preneoplastic processes^23^, the Atf4^high^/GFP^+^ cells may be the source of gastric cancer via transdifferentiation into SPEM-like cells.

## Methods

### Generation of inducible H2b-GFP knock-in mice

Generation of inducible H2b-GFP knock-in mice was performed by using CRISPR/Cas9^43^. In brief, a mixture containing a sgRosa26-1 crRNA^43^ (8.7 ng/μl, Fasmac, Japan), a tracrRNA (14.3 ng/μl, Fasmac, Japan), a single strand oligo donor nucleotide (ssODN) composed of 5′ arm, adenovirus splicing acceptor, SV40 pA, TRE3G promoter, H2b-GFP fusion cDNA, bovine growth hormone pA, EF1a promoter, Tet-on-3G sequences, rabbit beta globin pA, and 3′ arm (10 ng/μl, Fasmac, Japan), and Cas9 protein (80.5 ng/μl, New England Biolabs Japan) was microinjected into pronuclei of fertilized eggs of C57BL6/N mice. All animal care and experimental procedures were approved by animal ethics committees of National Cancer Center Research Institute (A410-19) and Tokyo Women’s Medical University (AE20-042-3), and we have complied with all relevant ethical regulations and guidelines for animal use.

### Validation of CRISPR-based knock-in of inducible H2b-GFP

Mouse tails were excised and lysed in 0.1 ml SNET buffer (20 mM Tris-HCl pH 8.0, 5 mM EDTA, 400 mM NaCl, 0.3% SDS) containing 0.02 mg/ml Proteinase K (Promega, MC5005) at 55 °C for 6 h. The lysates were diluted 100-fold with DNA suspension buffer (TEKnova, T0227), and 1 µl of the diluents was subjected to PCR (30 cycles of 98  °C for 10 s, 60  °C for 5 s, and 68 °C for 20 s) followed by an extension at 68 °C for 1 min in 20 μl buffer containing 1x KOD one DNA polymerase master mix (Toyobo, KMM-201), 200 nM forward primer, and 200 nM reverse primer. The sequence of the primers shown in Supplementary Fig. 1a are as follows: Rosa26-F: 5′- CAAGCACGTTTCCGACTTGA-3′, Rosa26-R: 5′- CCAATGCTCTGTCTAGGGGT-3′, Ins-F: 5′- TTCGTGAACGACATCTTCGAGCGC-3′, Ins-R: 5′- GTCTTGTAGTTGCCGTCGTCCTTG-3′.

### Doxycycline treatment

Four-week-old Rosa26^iH2b-GFP^ mice were orally administered with doxycycline-containing diet (200 mg/kg, Research Diets, Inc., Japan) for 4 weeks and then fed with standard diet without doxycycline for the indicated chase periods.

### Immunofluorescence

Gastrointestinal tissues were fixed in 10% formaldehyde overnight at 4  °C, embedded in paraffin, sliced into 4 μm sections, and stained with hematoxylin and eosin for histological examination. For immunofluorescent analyses, slides were subjected to antigen retrieval with 10 mM citrate buffer (pH 6.0) and stained with the following primary antibodies: rabbit anti-GFP (1:100; Cell Signaling, #2555); mouse anti-GFP (1:50; Santa cruz, sc-9996); goat anti-Cdh1 (1:100; R&D Systems, AF748); rabbit anti-Mki67 (1:100; Cell Signaling, #12202); mouse anti-Krt19 (1:50; Santa cruz, sc-6278); rabbit anti-Dclk1 (1:100; Abcam, ab109029); rabbit anti-Chromogranin A (1:100; Thermo Fisher scientific, #RB-9003); rabbit anti-Pla2g1b (1:100; Invitrogen, #PA5-96336); rabbit anti-Chia1 (1:100; Proteintech, 21484-1-AP); rabbit anti-Troy (1:100; Invitrogen, MA5-36190); rabbit anti-Atf4 (1:100; Bioss, bs-1531R); rabbit anti-Hspa8 (1:100; Abcam, ab51052); and rabbit anti-Furin (1:100; Cell Signaling, #04709). Subsequently, the slides were stained with the following secondary antibodies: donkey anti-goat IgG Alexa Fluor 555-conjugated (1:1000; Invitrogen, A21432); donkey anti-mouse IgG Alexa Fluor 647-conjugated (1:1000; Invitrogen, A31571); donkey anti-mouse IgG Alexa Fluor 750-conjugated (1:1000; Abcam, ab175739); donkey anti-rabbit IgG Alexa Fluor 647-conjugated (1:1000; Invitrogen, A31573); and donkey anti-rabbit IgG Alexa Fluor 750-conjugated (1:1000, Abcam, ab175728). For sequential immunostaining with primary antibodies from identical host species (i.e., double staining of Mki67 with Pla2g1b or Atf4), first-round staining was performed by using the Alexa Fluor 488 tyramide reagent (Invitrogen, B40953) and the VECTASTAIN Elite ABC Kit (Vector Laboratories, PK-6101) according to the manufactures’ protocols. Subsequently, the antibodies were removed by heating in a pressure cooker in 10 mM citrate buffer (pH 6.0) for 10 minutes, and then the slides were subjected to second-round staining. Nuclear DNA was counterstained with 10 µg/ml Hoechst 33342 (Invitrogen, H3570). Immunofluorescent images were examined with a fluorescent microscope (BZ-X810, Keyence).

### FACS sorting of gastrointestinal tissues

Stomach, small intestine, and colon tissues were obtained from doxycycline-treated or untreated mice, minced with razor blades, and incubated 45 min at 37 °C in HBSS (Gibco, #14025-092) supplemented with 3 mg/ml collagenase P (Roche, #11213865001), 1 mg/ml Dispase II (Roche, #04942078001), and 1 µg/ml DNase I (Roche, #10104159001). The dissociated cells were washed twice with FACS buffer [D-PBS(−) containing 0.5% BSA and 0.5 mM EDTA], and incubated with Accumax reagent (Innovative Cell Technologies, AM-105) for 15 min at 37 °C. Subsequently, cells were subjected to gentle pipetting and filtration with a 100 µm filter.

The enzymatically digested samples were subjected to antibody staining for 30 min on ice with anti-mouse Cd31 antibody conjugated with APC (1:300; Biolegend, #102410), anti-mouse Cd45 antibody conjugated with APC (1:300; Biolegend, #103112), and anti-mouse Epcam antibody conjugated with PE (1:300; eBioscience, #12-5791-82). After immunostaining, cells were subjected to Calcein Blue AM viability dye staining at 37 °C for 15 min according to the manufacturer’s protocol (Invitrogen, #65-0855-39). The stained cells were collected by centrifugation, re-suspended with FACS buffer, combined with TO-PRO3 Ready Flow reagent (Invitrogen, R37170), and subjected to sorting by FACS AriaIIIu (Becton Dickinson). Viable cells were selected as Calcein-blue^+^/TO-PRO3^-^. Hematopoietic cells and endothelial cells were removed by staining with Cd45 and Cd31, respectively. Epithelial cells were selected as Epcam-positive cells (Epcam^+^ cells), and GFP label-retaining epithelial cells were selected as GFP-positive cells within the Epcam^+^ cell population (GFP^+^ cells).

### Single-cell RNA-seq

Stomach tissues of doxycycline-treated female Rosa26^iH2b-GFP^ mice were isolated 6 weeks after the beginning of chase periods and subjected to epithelial cell isolation and FACS sorting for Epcam^+^ and GFP^+^ cells. The sorted cells from two female mice were combined and loaded onto a Chromium next GEM chip G (PN-2000177, 10x Genomics) and captured into Gel Beads-in-emulsion (GEMs) by using a Chromium Controller (10x Genomics). cDNA synthesis and sample-indexed library construction were performed using a Chromium next GEM single-cell 3’ reagent kit (PN-1000121, 10x Genomics) according to the manufacturer’s protocol. Libraries were subjected to sequencing on a HiSeq2500 (Illumina). Mapping of raw sequence reads to mm10 mouse reference genome (version 2020-A, provided by 10x Genomics) and the initial quality control was performed using Cell Ranger software v3.0.2 (10x Genomics). The gene count matrices were imported to Seurat software v4.1.1 running on R v4.1.2. Cell meeting the following criteria were removed from dataset: cells with > 15% of mitochondrial gene counts; cells with >8000 unique featureCounts; cells with <1500 unique featureCounts; cells with positive counts for *Ptprc* (*Cd45*) or *Pecam1* (*Cd31*) genes. In total, 4449 cells were subjected to further analyses.

### Single-cell RNA-seq data analysis

Analyses of the filtered gene count matrices were performed by Seurat pipeline (https://satijalab.org/seurat/articles/pbmc3k_tutorial.html). The single-cell gene expression counts were log-normalized by using NormalizeData() with a scaling factor of 10,000. The top 2000 most variable genes were identified by FindVariableFeatures(), scaled with ScaleData(), and subjected to principal component analyses to determine top principal components. Subsequently, the top principal components were subjected to Uniform Manifold Approximation and Projection (UMAP) dimensionality reduction by RunUMAP(), which was subjected to graph-based cluster detection and visualization using DimPlot. Gene expression levels were represented as violin plots using VlnPlot() or feature plots by using FeaturePlot(). Differentially expressed genes (DEGs) for each cluster were identified by Wilcoxon’s signed-rank test using FindAllMarkers(). A *p* value (<0.05) and a log2 fold change (log2FC) cutoff (>0.25) were used to select DEGs. Co-expression of quiescent cell markers in the 1a subpopulation was examined by correlation coefficients and hierarchical clustering by Spearman method and ward-D2 method in corrplot (v0.92), respectively.

### Enrichment analyses

DEGs (a Bonferroni-adjusted *p* value < 0.05) between Epcam^+^ and GFP^+^ cells in cluster 1 chief cells were selected by using FindAllMarkers(), and GO enrichment analyses were performed by using clusterProfiler v4.2.2 to identify the top ten most significant GO terms in the biological processes (BP) and molecular functions (MF) categories. Gene concept networks were generated to depict the linkage of genes of selected GO terms by using centplot(). ssGSEA for single-cell RNA-seq data was performed using escape v1.5.1. The statistical significances of ssGSEA were calculated using getSignificance().

### Quantification of transcription factor activities

The inferred activities of major transcription factors in each cell were determined using VIPER (Virtual Inference of Protein-activity by Enriched Regulon analysis) v1.28.0., which is based on a curated collection of transcription factors and their target genes (DoRothEA v1.6.0). Transcription factor-target interactions classified as confidence levels A and B were exclusively used as high-quality regulons. VIPER scores were used for split violin plots, feature plots, or heatmaps using pheatmap v1.0.12.

### Indomethacin-induced stomach injury

Doxycycline-treated Rosa26^iH2b-GFP^ mice at 6 weeks after the beginning of chase periods were subjected to fasting overnight with free access to water, and then orally administrated with 20 mg/kg indomethacin in 0.5% carboxymethylcellulose and 20% ethanol. The stomachs of the indomethacin-treated mice after 6 h (*n* = 4) and 48 h (*n* = 4) of indomethacin administration were subjected to histological examination and immunofluorescence. Non-treated mice were used as control (*n* = 4). The numbers of GFP^+^ and GFP/Mki67^+^ cells within the bottom third of the corpus epithelia were counted to evaluate the emergence of GFP^+^ chief cells initiating proliferation.

### Statistics and reproducibility

Data are represented as mean ± standard deviation. For calculation of *p* values, two-tailed Student’s *t* test was performed except comparison of data shown in Supplementary Fig. 4e, for which the Wilcoxon test was used for the calculation. The number of replicates was described in figure legends.

### Supplementary information

Supplementary Information

Description of additional supplementary files

Supplementary Data 1

## Data Availability

The RNA-seq data are available at the Gene Expression Omnibus (GEO) database with accession number GSE213755. Numerical data underlying all graphs and plots in the manuscript can be found in Supplementary data file. All the other data within the article and its Supplementary Information are available from the corresponding authors.
