# Supplementary material for: Elevated stress response marks deeply quiescent reserve cells of gastric chief cells
Source: Commun Biol. 2023 Nov 20;6:1183. doi: 10.1038/s42003-023-05550-2 (PMC10662433; doi:10.1038/s42003-023-05550-2)
Supplement: Supplementary file 1 — Supplementary Information [file 42003_2023_5550_MOESM1_ESM.pdf]

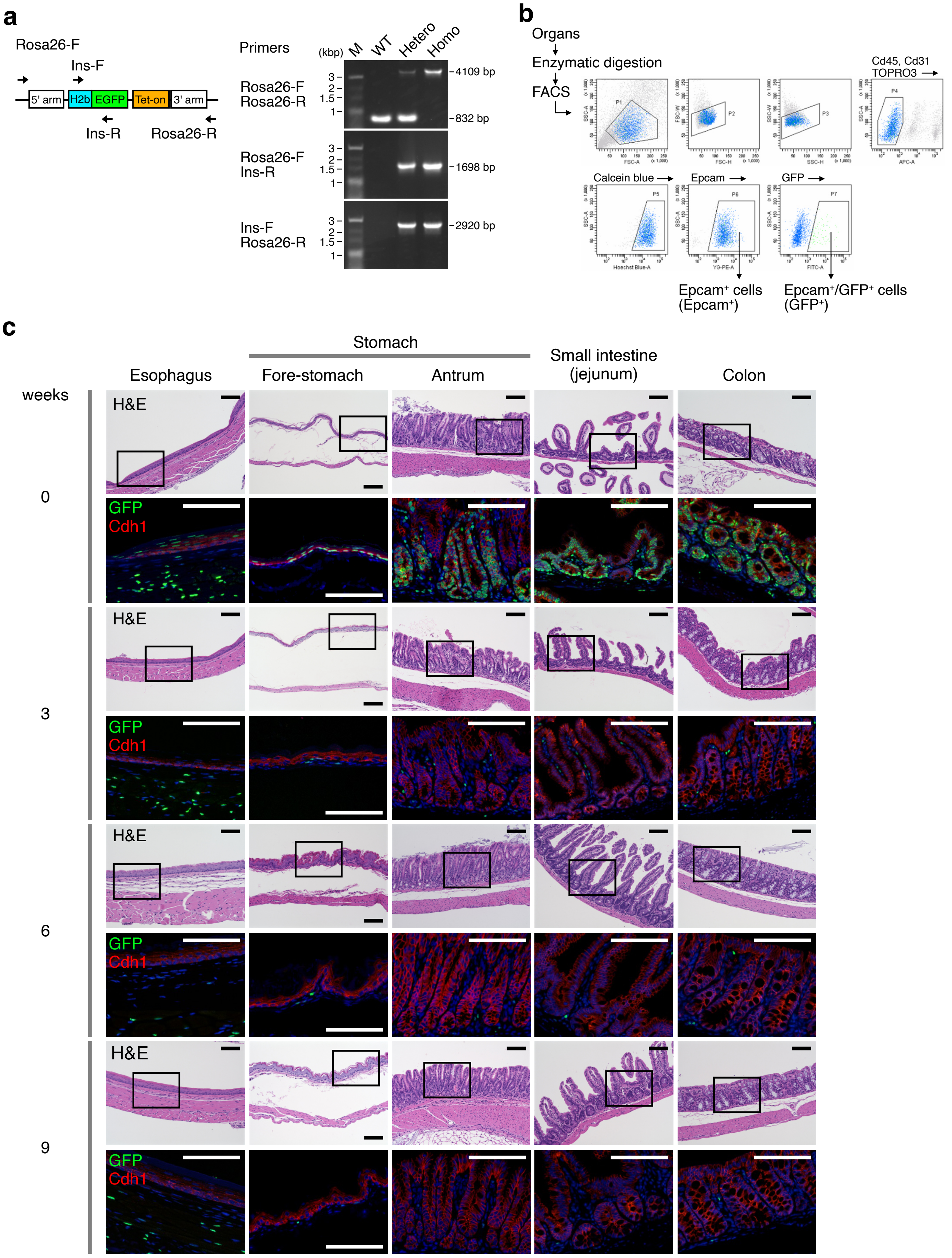

Supplementary Figure 1

Supplementary Fig. 1 Stomach epithelia specifically harbor label-retaining long-term quiescent cells. **a** Validation of integration of the inducible H2b-GFP cassette at the *Rosa26* loci. (left) a schematic illustration of positions and directions of primers used to examine cassette integration. (right) PCR amplification of genomic fragments from wild type (WT), Rosa26$^{wt/iH2b\text{-}EGFP}$ (Hetero), and Rosa26$^{iH2b\text{-}EGFP/iH2b\text{-}EGFP}$ (Homo) mice. M, size markers. **b** Experimental scheme for FACS sorting of Epcam^{+} cells and GFP^{+} cells. **c** H&E (upper panel) and immunostaining (lower panel) of the H2b-GFP^{+} cells in gastrointestinal tissues (esophagus, forestomach, antrum, small intestine, and colon) at the indicated times (weeks) after the beginning of chase periods. Black boxes indicate the regions shown in immunostaining. Scale bar, 100 μm.

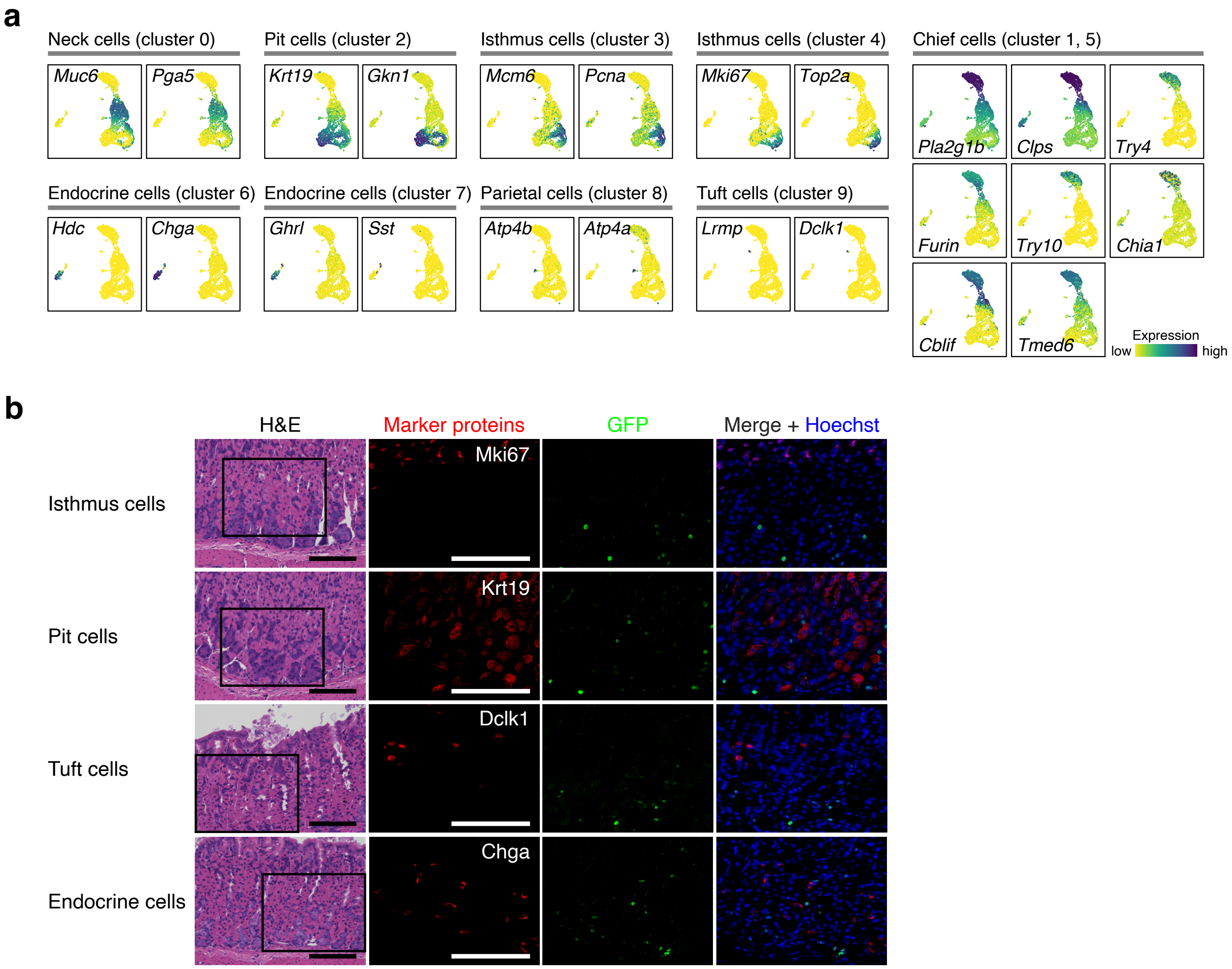

Supplementary Figure 2

Supplementary Fig. 2 Long-term quiescent cells in the stomach are localized to the gastric corpus. **a** Expression levels of the indicated marker genes in the stomach epithelial cells shown in UMAP Feature plots. **b** H&E and immunostaining of the H2b-GFP$^+$ cells of stomach corpus at 6 weeks after the beginning of chase periods. Black boxes in the H&E images show the regions corresponding to the immunostainings (the indicated marker proteins and GFP). Black boxes indicate the regions shown in immunostaining with the indicated antibodies. Scale bar, 100 μm.

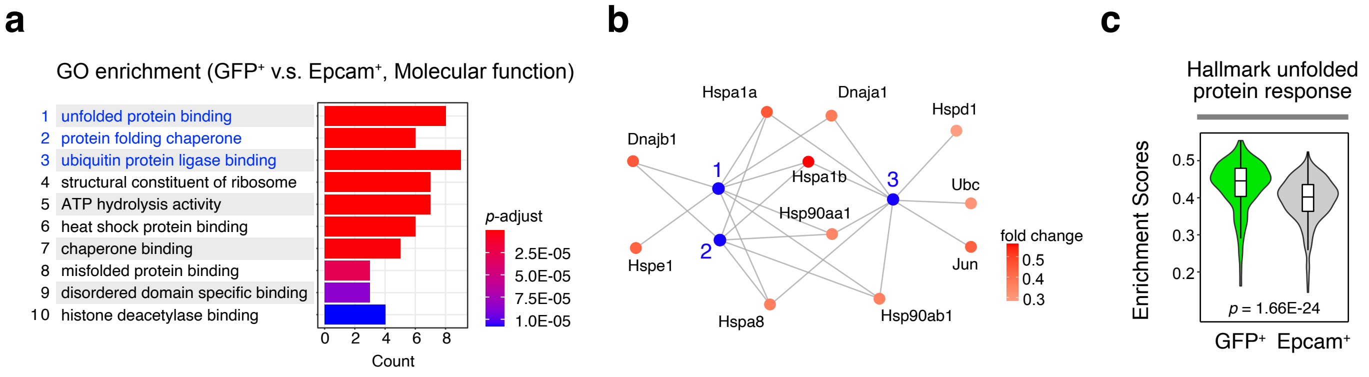

Supplementary Figure 3

Supplementary Fig. 3 Atf4 and the unfolded protein response pathway are activated in deeply quiescent chief cells. **a** Gene Ontology (GO) term enrichment analyses (Molecular function) of GFP$^+$ chief cells. The top ten GO terms enriched in GFP$^+$ cells in comparison to Epcam$^+$ cells are shown. The *p*-values and gene counts of top 10 GO terms enriched in GFP$^+$ cells in comparison to Epcam$^+$ cells are shown. The top three terms were shown in blue characters. *p* value of each GO term is color labeled. **b** Gene-concept network for top three GO terms. Blue dots represent the top three terms shown **a** (1, unfolded protein binding: 2, protein folding chaperone: 3, ubiquitin protein ligase binding). **c** Violin plots of ssGSEA enrichment scores (unfolded protein response). Calculation of the enrichment score was performed by using GFP$^+$ (402 cells) and Epcam$^+$ cells (494 cells) of the cluster 1 population.

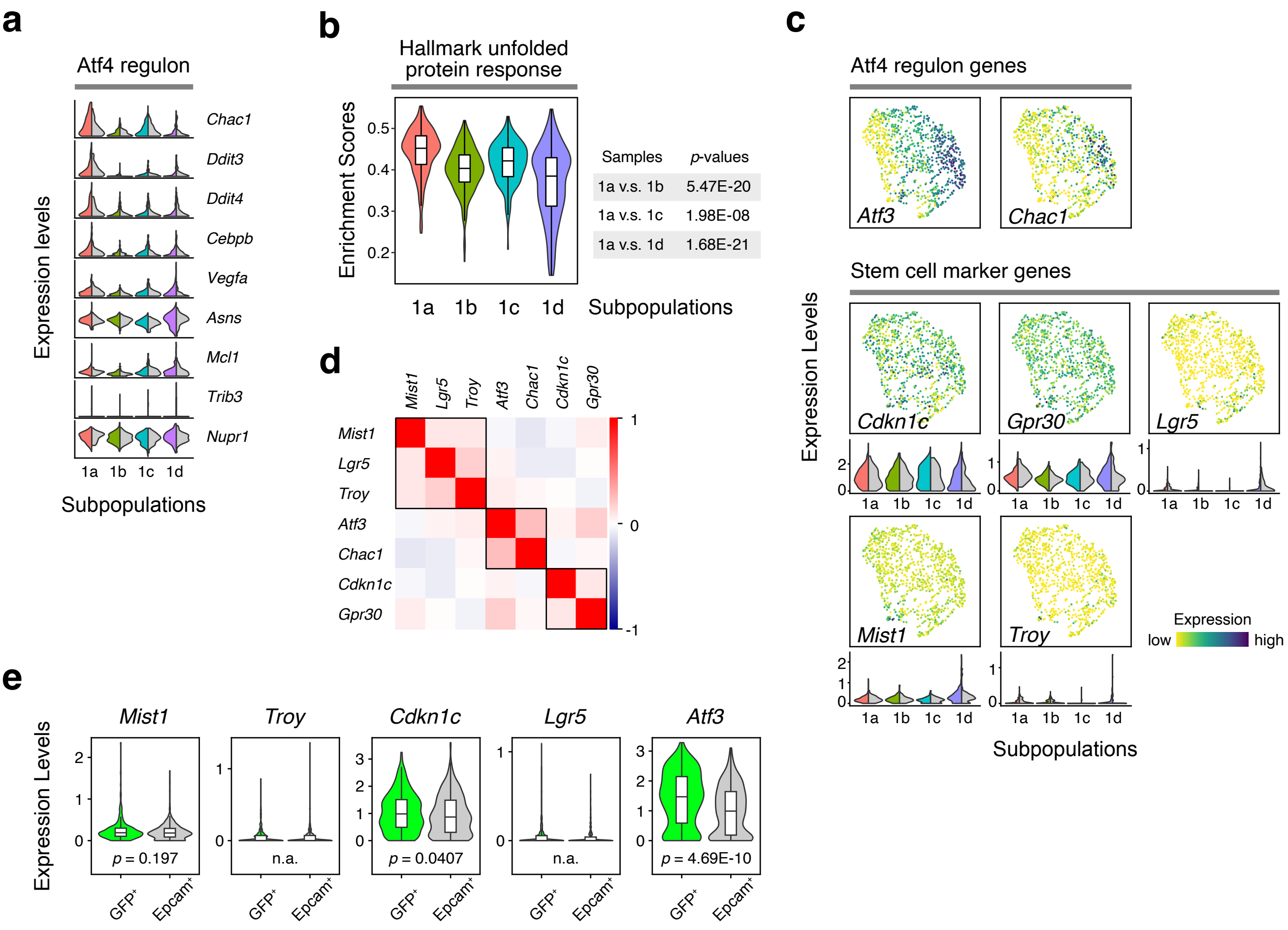

Supplementary Figure 4

Supplementary Fig. 4 Atf4^{high} quiescent cells comprise a unique subpopulation of chief cells. **a** Split violin plots for the Atf4-regulated genes organizing the Atf4 regulon (left: mixture of Epcam^{+} cells and GFP^{+} cells, right: Epcam^{+} cells). **b** Violin plots of ssGSEA enrichment scores (unfolded protein response). Calculation of enrichment score was performed using the indicated subpopulation of cluster 1 (numbers of cells: 1a = 279, 1b = 260, 1c = 181, 1d = 176). **c** Expression levels of the indicated stem cell marker genes in c1 chief cells shown in UMAP feature plots (upper panels) and split violin plots (lower panels). (left: mixture of Epcam^{+} cells and GFP^{+} cells, right: Epcam^{+} cells). **d** A correlation matrix for known quiescent markers (*Mist1*, *Lgr5*, *Troy*, *Cdkn1c*, *Gpr30*) and Atf4 target genes (*Atf3*, *Chac1*) in the c1 chief cells. **e** Violin plots of the indicated chief cell markers and *Atf3* in cluster 1 GFP^{+} cells (402 cells) and Epcam^{+} cells (494 cells).
