# Supplementary material for: Elevated stress response marks deeply quiescent reserve cells of gastric chief cells
Source: Commun Biol. 2023 Nov 20;6:1183. doi: 10.1038/s42003-023-05550-2 (PMC10662433; doi:10.1038/s42003-023-05550-2)
Supplement: Supplementary file 2 — Description of additional supplementary files [file 42003_2023_5550_MOESM2_ESM.docx]

Description of Additional Supplementary Files

**File name:** Supplementary Data 1

**Description:** The source data behind the graphs in the paper.
